# Near-complete genome sequence of *Cydia pomonella* granulovirus BZR GV 10, a potential biocontrol agent against apple codling moth

**DOI:** 10.1128/mra.00207-25

**Published:** 2025-06-09

**Authors:** Aleksandra A. Tsygichko, Anzhela M. Asaturova, Gennady V. Vasiliev, Alexandra I. Klimenko, Sergey A. Lashin, Tatiana N. Lakhova

**Affiliations:** 1 Federal State Budgetary Scientific Institution, Federal Research Center of Biological Plant Protection, Krasnodar, Russia; 2 Kurchatov Genomic Center of Institute of Cytology and Genetics SB RAS, Novosibirsk, Russia; Katholieke Universiteit Leuven, Leuven, Belgium

**Keywords:** baculovirus, *Cydia pomonella*, bioinsecticides, genome analysis, CpGV

## Abstract

We report the near-complete genome of *Cydia pomonella* granulovirus BZR GV 10. The genome sequence is 123,362 bp long and shows a degree of similarity of more than 99% to the reference isolate Mexico/1963.

## ANNOUNCEMENT

*Cydia pomonella* granulovirus (CpGV) virus has a double-stranded DNA genome of approximately 123,500 bp. CpGV strains can be used as the active component in insecticides to control *C. pomonella* L., 1758 (1, 2). However, isolating and characterizing new effective strains is still of great importance, since the challenge of resistance to these lethal strains arises (3, 4). We present the near-complete genome sequences of a CpGV strain from the bioresource collection of the Federal Research Center of Biological Plant Protection «State Collection of Entomoacariphages and Microorganisms», Krasnodar, Russia.

The strain was isolated in the Rostov region of Russia in 2018. Total viral DNA was extracted from the *C. pomonella* larvae by centrifuge sedimentation using the PureLink Genomic DNA Mini Kit (Invitrogen) (5). DNA concentration was determined fluorimetrically on a Qubit device using the High Sensitivity kit. The KAPA DNA Prep kit was used to develop genomic libraries. The KAPA Unique Dual-Indexed Adapter Kit was used for barcoding. The libraries were amplified by nine cycles of PCR and purified by the addition of an equal volume of Agencourt AMPure XP. The quality of the libraries was determined using a BA2100 bioanalyzer. Sequencing was carried out on a NextSeq550 device with 2 × 150 bp paired-end reads according to the manufacturer’s protocol, resulting in 25,197,403 nucleotide read pairs.

FаstQC was used to check the quality of the acquired reads. Fastp was used for read-quality trimming (6). We mapped the reads against the reference genome of *C. pomonella* granulovirus isolate Mexico/1963 (NC_002816) from the NCBI RefSeq database. Mapping was performed by the BWA-MEM program, without regard to mapping quality (7). Two percent of the reads were successfully mapped (503,948 reads in total). The genome coverage after mapping is greater than 690×. We used these reads to assemble the genome with SPAdes to acquire the first draft assembly (8). We used an alternative approach employing the whole set of reads (not only mapped ones) while assembling with MinYS (9). Both near-complete assemblies were processed by Pilon (10) to improve the genome assembly by reducing the number of assembly errors. The final assembly was obtained using GFinisher (11) from these two near-complete assemblies (SPAdes: the main assembly and MinYS: the alternative). The quality of all interim and final assemblies was controlled using Quast (12). The resulting assembly was annotated with Prokka (13). A detailed set of programs used is described in reference 5. We analyzed the average nucleotide identity of the final assembly and the reference genome using FastANI (14).

The final BZR GV 10 assembly consists of three contigs, which are 123,362 base pairs in total (Table 1). Comparison of the strain BZR GV 10 genome with the reference genome (NC_002816) shows a nucleotide similarity of 99.8%.

**TABLE 1:** The key metrics for each genomic segment of the BZR GV 10 strain

Key metrics	Genome segments		
	OR743693	OR743694	OR743695
CG content (%)	41.79	45.44	45.18
Average depth	811.97	767.56	743.48
Segment length (bp)	2,168	51,719	69,475
Genomic coverage (%)	1.65	41.898	56.246
Number of ORFs	2	61	74

## Data Availability

Near-complete genome of the BZR GV 10 strain (CpGV) obtained during the study has been deposited in GenBank under accession numbers OR743693, OR743694, and OR743695. The initial NGS data are presented in the SRA NCBI: SRR29856645.
